# Clinical and economic impact of changing reimbursement criteria for statin treatment among patients with type 2 diabetes mellitus in South Korea

**DOI:** 10.3389/fphar.2022.924141

**Published:** 2022-08-30

**Authors:** Siin Kim, Kyungseon Choi, Ji-yool Kim, Hae Sun Suh

**Affiliations:** ^1^College of Pharmacy, Kyung Hee University, Seoul, South Korea; ^2^ Viatris Korea Ltd., Seoul, South Korea

**Keywords:** cardiovascular event, diabetes, economic impact, reimbursement criteria, statin

## Abstract

**Aim:** Patients with type 2 diabetes mellitus (T2DM) in South Korea can be reimbursed for statins if they have a low-density lipoprotein cholesterol (LDL-C) level of ≥100 mg/dL. We aimed to explore the clinical and economic benefit received by T2DM patients when easing the current criteria for statin treatment by lowering the LDL-C threshold from 100 mg/dL to 70 mg/dL.

**Methods:** We used a static course model with a 5-year period to compare the following two scenarios in T2DM patients with no history of cardiovascular (CV) events: the current criteria covering LDL-C ≥100 mg/dL and the revised criteria covering LDL-C ≥70 mg/dL. The number of target patients was estimated based on previous Korean studies on patients with T2DM. The current mix of treatments used for T2DM and costs involving CV events were estimated using the National Health Insurance Service–National Health Screening Cohort database. The baseline CV event rates and case fatality were estimated using NHIS Customized database, including 50% patients who were prescribed atorvastatin and 100% who were not prescribed statins between 2009 and 2012 among patients with T2DM in the entire Korean population. After propensity score matching, patients with T2DM not prescribed statins were followed up until 2018 to estimate the incidence rates of coronary heart disease (CHD) and stroke. The efficacy of atorvastatin for the primary prevention of CV events in patients with T2DM was derived from a pivotal clinical trial. The outcome measures were the number of CV events prevented after the change in criteria and the consequent cost savings.

**Results:** In South Korea, the current and revised criteria covered 2,434,379 and 3,446,149 patients with T2DM, respectively. The change in criteria resulted in the prevention of 726 CV events and cost savings of US dollars (USD) 5.5 million at the national level and USD 0.0089 per member per month in the fifth year.

**Conclusion:** Easing the reimbursement criteria for statin treatment among patients with T2DM was associated with a reduction in CV events and their related costs; therefore, changing the reimbursement criteria is worth further consideration to mitigate the burden of CV disease.

## 1 Introduction

Cardiovascular disease (CVD) is associated with an enormous burden, including a high mortality rate and healthcare cost, worldwide (Leal et al., 2006; Deaton et al., 2011; Fox et al., 2016). Elevated low-density lipoprotein cholesterol (LDL-C) levels are a major risk factor for CVD and are associated with an increased risk of cardiovascular (CV) events and CVD-associated mortality (Pekkanen et al., 1990; Wong et al., 1991; Wilson et al., 1998). Therefore, statin therapy is recommended for the management of LDL-C levels in patients with CVD or those at high risk of CVD (Grundy et al., 2019). Patients with type 2 diabetes mellitus (T2DM) are considered to be at a high risk of CVD, which accounts for 20–49% of the total healthcare cost of T2DM (Einarson et al., 2018).

As T2DM and CVD are the leading causes of the burden of illness in South Korea (Oh et al., 2011; Jung et al., 2021), LDL-C management is recommended based on LDL-C goals stratified by patient risk levels (Kim et al., 2016). In South Korea, the reimbursement criteria for statin treatment are also based on patients’ LDL-C levels and risk levels and can be summarized as follows: 1) LDL-C ≥70 mg/dL in patients with acute coronary syndrome; 2) LDL-C ≥100 mg/dL in patients with coronary artery disease (CAD) or equivalent risk (i.e., diabetes mellitus, peripheral artery disease, abdominal aortic aneurism, and carotid disease); 3) LDL-C ≥130 mg/dL in patients with two or more risk factors (i.e., smoking, hypertension, HDL-C <40 mg/dL, family history of premature CAD, and age ≥45 years in men and ≥55 years in women); and 4) LDL-C ≥130 mg/dL in patients with one or no risk factors (Health Insurance Review and Assessment Service, 2014). Accordingly, patients with diabetes can be reimbursed for statin treatment if they have LDL-C levels ≥100 mg/dL.

Several previous studies have suggested the benefit of intensive treatment goals in preventing CV events among patients with T2DM with relatively low LDL-C levels, such as those with <100 mg/dL (Fleg et al., 2008; Itoh et al., 2018; Wang et al., 2020; Shinohara et al., 2021). In a phase 3 randomized clinical trial of atorvastatin treatment in patients with T2DM with no history of CVD, a post-hoc analysis revealed that atorvastatin was associated with a 26% reduction in CV events among patients with baseline LDL-C <100 mg/dL (Colhoun et al., 2004). Moreover, the American College of Cardiology/American Heart Association has suggested approaches for lipid management based on risk reduction benefits rather than specific LDL-C goals, recommending that adult patients with T2DM start statin treatment regardless of their LDL-C levels (Stone et al., 2014; Grundy et al., 2019).

Considering the existing evidence, statin treatment in a broader group of patients with T2DM, including those with relatively low LDL-C levels, might have an additional benefit regarding the prevention of CVD and its related costs. Therefore, we assessed the clinical and economic benefit of CV event prevention when easing the reimbursement criteria for statin treatment in patients with T2DM in South Korea.

## 2 Methods

### 2.1 Study design and data source

We estimated the clinical and economic impact of changes in reimbursement criteria using a static course model with a 5-year period from a Korean payer’s perspective, assuming that the epidemiology of the disease remains unchanged over time. Two reimbursement scenarios were compared: current criteria, covering patients with T2DM with LDL-C ≥100 mg/dL and revised criteria, covering patients with T2DM with LDL-C ≥70 mg/dL. We assumed that atorvastatin is the only drug used in both scenarios, given that only atorvastatin and simvastatin are indicated for diabetes, and the prescription of atorvastatin is far more frequent than that of simvastatin in South Korea (Kim and Rhew, 2021). The study population included patients with T2DM with LDL-C ≥70 mg/dL and no previous history of CV events.

The input parameters used in this model are listed in Table 1. Most input values were based on the National Health Insurance Service (NHIS) database and published literature. We utilized two types of NHIS databases: the NHIS-National Health Screening Cohort (NHIS-HEALS) database and the NHIS Customized database. The former includes information on sociodemographic characteristics, healthcare resource use, disease diagnosis, health screening results, and death during 2002–2015, which was collected from a 10% random sample of all patients who participated in health screening during 2002–2003, assuring the representativeness of laboratory test results, such as LDL-C (Seong et al., 2017). Having a data structure similar to that of the NHIS-HEALS, the NHIS Customized database consists of data on diabetes patients among the entire Korean population between 2009 and 2012, with 50% diabetes patients who were prescribed atorvastatin and 100% with no statin prescriptions. We were able to obtain 50% of the sample rather than all patients with diabetes who were prescribed atorvastatin because this was the maximum sample that could be utilized under the limited data size imposed by the NHIS. The data period of the NHIS Customized database was 2008–2018, and patients with cancer diagnoses (International Classification of Diseases, 10th revision [ICD-10] codes of C00–C97) from 2008 to 2018 were excluded from the database. In this study, we utilized the NHIS Customized database to estimate the efficacy of atorvastatin in the primary prevention of CV events, given that this database contains the largest sample of the population of interest and appropriate controls. However, the patients included in the database were not representative of all patients with diabetes in Korea but were limited to a subgroup of patients with diabetes. Therefore, we estimated the current mix of treatments used for T2DM and CV event costs using the NHIS-HEALS database, which provided a 10% sample of health-screening participants in Korea.

**TABLE 1:** Input parameter values and data sources used in the model.

Parameter	Value	Source
Population		
Total Korean population in 2020	51,780,579	KOSIS
Prevalence of T2DM, %	8.4	Koo et al. (2014)
Proportion of patients without previous CV events among patients with T2DM, %	89.2	Rhee et al. (2011)
LDL-C distributions of patients with T2DM, %		Kim et al. (2019)
70–99 mg/dL	26.1	
≥100 mg/dL	62.7	
The current mix of treatments		NHIS-HEALS DB
Proportion of patients taking atorvastatin, %	11.6	
CV event costs, USD		NHIS-HEALS DB
Non-fatal CHD	7,440	
Fatal CHD	4,674	
Non-fatal stroke	8,057	
Fatal stroke	4,802	
Efficacy		
Baseline CV event rates, per 100 PYs		NHIS Customized DB
CHD	0.59	
Stroke	0.85	
Hazard ratio of CV events		Colhoun et al. (2004)
CHD	0.640	
Stroke	0.520	
Case fatality, %		NHIS Customized DB
CHD	12.2	
Stroke	5.5	

CHD, coronary heart disease; CV, cardiovascular; DB, database; KOSIS, Korean Statistical Information Service; LDL-C, low-density lipoprotein cholesterol; NHIS, National Health Insurance Service; NHIS-HEALS, National Health Insurance Service–National Health Screening cohort; PYs, person-years; T2DM, type 2 diabetes mellitus; USD, United States Dollar.

### 2.2 Population

To estimate the number of target patients in the current and revised reimbursement criteria, we started from the entire Korean population and narrowed them down to the target population of the reimbursement criteria. The number of T2DM patients in South Korea in 2020 was estimated by applying the prevalence of T2DM estimated from a previous study using the Health Insurance Review and Assessment Service data (Koo et al., 2014). The proportion of patients with T2DM with no history of CVD was derived from the results of the Korean National Diabetes Program cohort (Rhee et al., 2011). The LDL-C distributions among patients with T2DM with no history of CVD were derived from a previous study using the NHIS Customized database, which reported the proportion of patients with LDL-C <70 mg/dL, 70–99 mg/dL, 100–129 mg/dL, 130–159 mg/dL, and ≥160 mg/dL as 11.2, 26.1, 31.5, 20.4, and 10.9%, respectively (Kim et al., 2019). Because we used a static course model, the number of target patients was assumed to be the same for the 5-year period.

### 2.3 The mix of treatments

**FIGURE 1:**
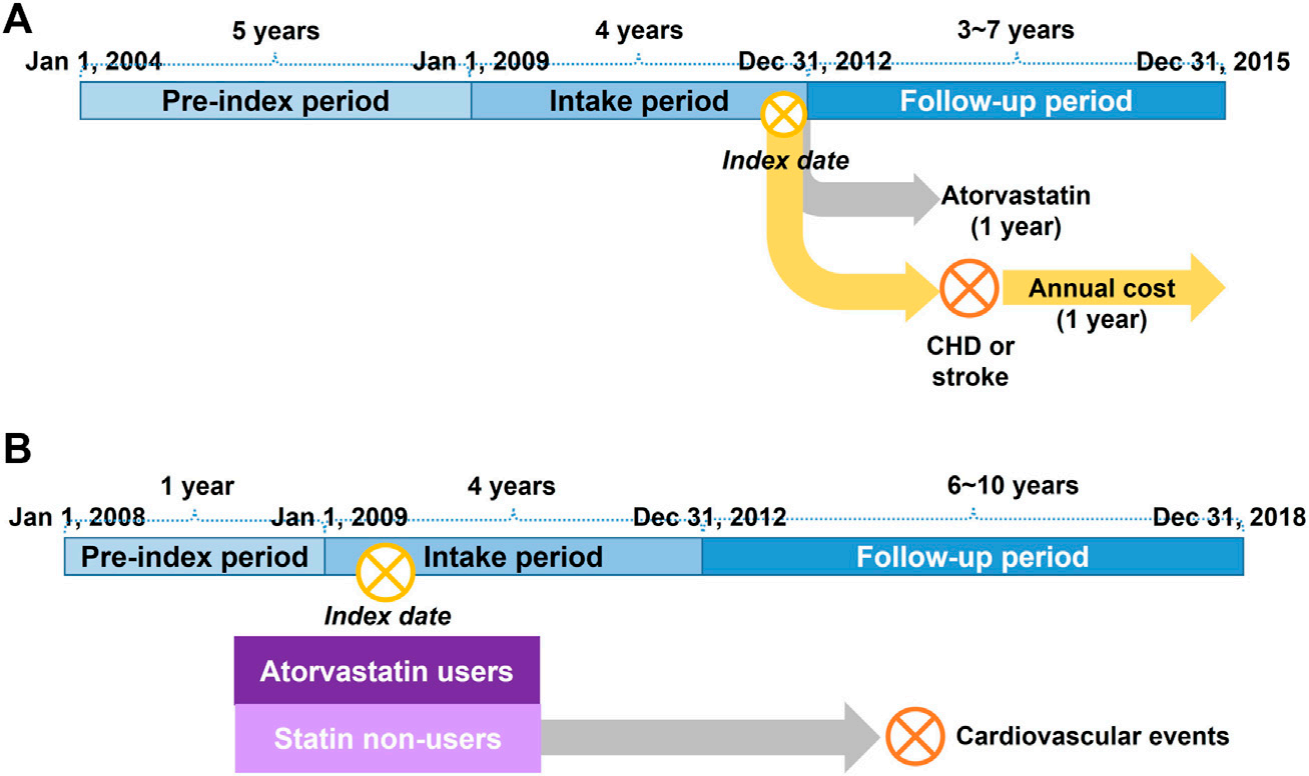
**(A)** Schematic diagram of the analysis of treatment mix and cost of cardiovascular events using the NHIS-HEALS database **(B)** Schematic diagram of the analysis of baseline cardiovascular event rates and case fatality using the NHIS Customized database. CHD, coronary heart disease; NHIS, National Health Insurance Service.

We analyzed the NHIS-HEALS database to estimate the proportion of patients prescribed atorvastatin among patients with T2DM with LDL-C levels ≥100 mg/dL (Figure 1A). Patients satisfying all of the following criteria were selected: 1) LDL-C ≥100 mg/dL at the first health examination they received between 1 January 2009 and 31 December 2012; 2) diagnosed with T2DM (ICD-10 codes E11, E14, and E14 up to the fifth position) and prescribed anti-diabetics within 1 year before the index date; 3) no history of lipid-lowering therapy, such as statins, ezetimibe, fibrates, nicotinic acid, and omega-3-acid ethyl esters, within 1 year before the index date; and 4) no history of CV events within 5 years before the index date (Table 2). The index date was defined as the first date of receiving a health examination between 1 January 2009, and 31 December 2012. The definition of CV events was based on previous studies that validated CV events in Korean national health insurance claims data (Kimm et al., 2012; Park and Choi, 2016). Eligible patients were followed-up for 1 year from the index date. As a result, 11.6% of the patients were prescribed atorvastatin.

**TABLE 2:** Diagnosis codes of cardiovascular events.

Cardiovascular event	ICD-10 code^*^	Description	Source
Myocardial infarction	I21	Acute myocardial infarction	Kimm et al. (2012)
Unstable angina	I20.0	Unstable angina	
Cardiac arrest	I46	Cardiac arrest	
Stroke	G46	Vascular syndromes of brain in cerebrovascular diseases	Park and Choi, (2016)
	I60	Subarachnoid hemorrhage	
	I61	Intracerebral hemorrhage	
	I63	Cerebral infarction	
	I64	Stroke, not specified as hemorrhage or infarction	

ICD-10, International Classification of Diseases, 10th revision; *In any position.

Because the NHIS-HEALS database includes only claims data generated under the current reimbursement criteria, we were unable to measure the proportion of patients prescribed atorvastatin among patients with diabetes with LDL-C <100 mg/dL. Therefore, the proportion of patients with LDL-C levels between 70 and 100 mg/dL was assumed to be the same as that of patients with LDL-C ≥100 mg/dL. Consequently, in the reimbursement scenario with the revised criteria, the proportion of patients with LDL-C between 70 and 100 mg/dL increased from 0 to 11.6% over the 5 years. This assumption was confirmed by an expert elicitation by an endocrinologist in South Korea.

### 2.4 Cost of CV events

The annual costs of coronary heart disease (CHD) and stroke were estimated using the same database as that of the treatment mix analysis (Figure 1A). We selected patients who underwent LDL-C examination between 1 January 2009, and 31 December 2012, with T2DM diagnosis and anti-diabetics within 1 year before the index date, and experienced CV events with hospitalization between the index date and 31 December 2014 (Table 2). The total medical costs incurred in 1 year from the first date of the CV event were measured from a Korean payer’s perspective. CV events were classified into fatal and non-fatal events, where fatal events were defined as cases of death within 28 days after the occurrence of a CV event (Xie et al., 2018). Costs were converted into US dollars (USD) using the average exchange rate in 2020 between Korean won and USD (1 USD = 1,180.11 Korean won).

### 2.5 Efficacy

The baseline CV event rates and case fatality among patients with T2DM who were not treated with statins were estimated from the NHIS Customized database (Figure 1B). First, patients treated with atorvastatin were selected according to the following criteria: 1) patients with an outpatient prescription with T2DM diagnosis (ICD-10 codes E11, E14, and E14 up to the fifth position), anti-diabetics, and atorvastatin between 1 January 2009, and 31 December 2012; 2) patients who were prescribed atorvastatin with a proportion of days covered ≥80% for 3 months after the index date; 3) patients with no history of atorvastatin in an outpatient setting within 1 year before the index date; 4) patients who received an LDL-C examination within 1 year before the index date; and 5) patients with no history of CV events within 1 year before the index date (Table 2). The index date was defined as the first date of outpatient prescription with T2DM diagnosis, antidiabetics, and atorvastatin between 1 January 2009, and 31 December 2012. Second, patients not treated with statins were selected according to the following criteria: 1) patients with an outpatient prescription with T2DM diagnosis and antidiabetics without any statin prescription between 1 January 2009, and 31 December 2012; 2) patients matched to the patients treated with atorvastatin by age and sex (up to 1:10 ratio); 3) patients who received an LDL-C examination within 1 year before the index date, the same as the index date of the matched atorvastatin users; and 4) patients with no history of CV events within 1 year before the index date. Lastly, patients treated with atorvastatin and those not treated with statins were matched using a 1:1 ratio according to the propensity scores. The propensity score was estimated by logistic regression, including age, sex, LDL-C, blood pressure, fasting blood sugar, smoking status, Charlson Comorbidity Index, and concomitant medication (antihypertensive and antiplatelet) as covariates. Greedy matching was conducted with calipers of 0.2 of the standard deviation of the logit of the propensity score (Austin, 2011). After propensity score matching, patients who were not treated with statins were followed up from the index date to the first date of CV events with hospitalization (Table 2), death, or 31 December 2018, whichever came first. The incidence rates of CHD and stroke were measured using the incidence density method. The case fatality of CHD and stroke was measured as the proportion of fatal events (i.e., death within 28 days) among all events (Xie et al., 2018). Further details of the analysis of the NHIS Customized database will be presented elsewhere (Kim and Suh, 2022).

The efficacy of atorvastatin in the primary prevention of CV events was derived from a pivotal clinical trial that compared atorvastatin with placebo among patients with T2DM without previous CV events (Colhoun et al., 2004).

### 2.6 Outcome measures

We measured the clinical and economic outcomes by comparing the two reimbursement scenarios. The clinical outcome was the number of CV events prevented after the change in the reimbursement criteria. The economic outcomes included total costs and per member per month (PMPM) costs resulting from the reduction in CV events. Total costs included annual medical costs related to CV events measured from a Korean payer’s perspective and were calculated at the national level by multiplying the annual cost of CV events per patient by the number of target patients in the reimbursement criteria. The PMPM costs were calculated on a monthly basis by dividing the total costs by the total Korean population in 2020.

### 2.7 Analyses

To determine the impact of uncertainty related to cost parameters, we conducted a sensitivity analysis by measuring the costs of CV events among patients with T2DM with no history of CV events within 5 years before the index date, whereas the costs were measured among all patients with T2DM regardless of CV event history in the base case. We also changed the baseline CV event rates based on published literature that assessed the risk of CV events according to LDL-C levels among patients with T2DM in South Korea (Kim et al., 2019). In the study, the baseline event rates of CHD and stroke in patients with LDL-C between 70 and 100 mg/dL were 0.32 and 0.56 per 100 person-years (PYs), respectively, and those in patients with LDL-C ≥100 mg/dL were 0.36 and 0.57 per 100 PYs, respectively.

An analysis with a static course model that assessed the clinical and economic impact of reimbursement criteria changes was conducted using Microsoft Excel (Microsoft, Redmond, WA, United States). The NHIS database was analyzed using the SAS Enterprise Guide 7.1 (SAS Institute Inc., Cary, NC, United States). This study was exempt from full review by the Institutional Review Board of Pusan National University (PNU IRB/2020_04_HR).

## 3 Results

### 3.1 Population size of the target patients in South Korea

Starting from the total Korean population in 2020, the patient group was narrowed to the target patients for the reimbursement criteria for statin treatment for T2DM (Table 3). Among patients with T2DM with no previous history of CV events, 1,011,770 had LDL-C levels between 70 and 100 mg/dL, and 2,434,379 had LDL-C levels ≥100 mg/dL. As a result, the current and revised criteria cover 2,434,379 and 3,446,149 patients, respectively, in South Korea. This change in reimbursement resulted in an approximately 40% increase in the number of patients with T2DM receiving reimbursement for statin treatment. When applying the mix of atorvastatin treatment to the target patients, 282,261 and 399,573 patients were estimated to be treated with atorvastatin before and after the change in reimbursement criteria, respectively.

**TABLE 3:** Calculating the population size of target patients of reimbursement criteria for statin treatment for type 2 diabetes mellitus.

Category	Value	Population size
Total Korean population in 2020	51,780,579	51,780,579
Prevalence of T2DM, %	8.4	4,349,569
Proportion of patients without previous CV events among patients with T2DM, %	89.2	3,879,815
LDL-C distributions of patients with T2DM, %		
70–99 mg/dL	26.1	1,011,770
≥100 mg/dL	62.7	2,434,379
Current criteria (covering LDL-C ≥100 mg/dL)		2,434,379
Revised criteria (covering LDL-C ≥70 mg/dL)		3,446,149

CV, cardiovascular; LDL-C, low-density lipoprotein cholesterol; T2DM, type 2 diabetes mellitus.

### 3.2 Clinical outcomes

**FIGURE 2:**
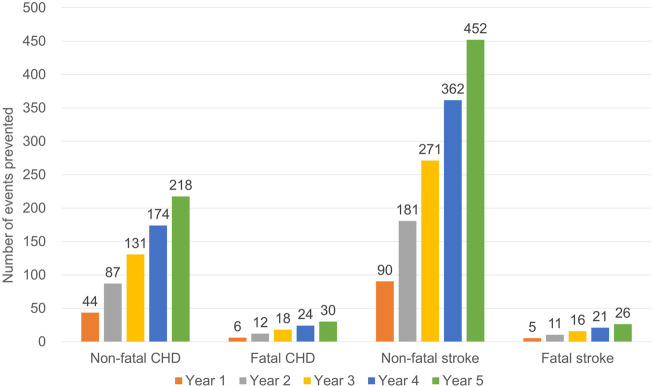
Predicted number of cardiovascular events prevented after the change in reimbursement criteria for statin treatment for type 2 diabetes mellitus. CHD, coronary heart disease.

A static course model estimated that 47,621 CV events occurred annually in the status quo among all patients with T2DM in South Korea (Table 4). After the change in reimbursement criteria, the number of CV events decreased to 46,895 in the fifth year after the change, resulting in the prevention of 726 CV events among all patients with T2DM in South Korea. Among the types of CV events, non-fatal stroke showed the largest number of events prevented followed by non-fatal CHD, fatal CHD, and fatal stroke (Figure 2).

**TABLE 4:** Clinical and economic outcomes before and after the change in reimbursement criteria for statin treatment for type 2 diabetes mellitus.

Category	Current criteria	Revised criteria				
		Year 1	Year 2	Year 3	Year 4	Year 5
Number of CV events	47,621	47,476	47,330	47,185	47,040	46,895
Non-fatal CHD	17,178	17,135	17,091	17,048	17,004	16,960
Fatal CHD	2,387	2,381	2,375	2,369	2,363	2,357
Non-fatal stroke	26,513	26,422	26,332	26,242	26,151	26,061
Fatal stroke	1,543	1,538	1,533	1,527	1,522	1,517
Total costs (USD)	359,984,234	358,878,362	357,772,491	356,666,619	355,560,747	354,454,875
PMPM costs (USD)	0.5793	0.5776	0.5758	0.5740	0.5722	0.5704

CHD, coronary heart disease; CV, cardiovascular; PMPM, per member per month; USD, United States Dollar

### 3.3 Economic outcomes

**FIGURE 3:**
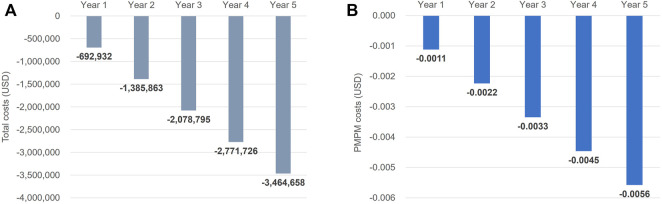
**(A)** Total cost savings and **(B)** per member per month cost savings resulted from the reduction of cardiovascular events after the change of reimbursement criteria for statin treatment for type 2 diabetes mellitus. PMPM, per member per month; USD, United States dollar.

The annual cost of CV events at the national level was estimated to be USD 360 million under the current criteria, and the reduction in CV events decreased the cost to USD 354 million in the fifth year after the change in the reimbursement criteria (Table 4). As a result, the estimated cost savings were USD 5,529,359 at the national level and USD 0.0089 PMPM in the fifth year (Figure 3).

In a sensitivity analysis using an alternative approach to measure the costs of CV events, which measured the costs among patients with T2DM with no history of CV events within 5 years, the cost of each CV event except for fatal CHD was slightly higher than that of the base-case analysis: USD 7,535, 4,375, 8,117, and 5,071 for non-fatal CHD, fatal CHD, non-fatal stroke, and fatal stroke, respectively. Consequently, the cost savings in the fifth year of change increased by <1% from the base-case analysis (USD 5,575,050 at the national level and USD 0.0090 PMPM in the fifth year of change). When changing the baseline CV event rates derived from published literature, the number of CV events prevented after the change in reimbursement criteria decreased compared to those of base-case analysis: 120, 17, 300, and 17 events for non-fatal CHD, fatal CHD, non-fatal stroke, and fatal stroke, respectively. This resulted in a 37% decrease in cost savings compared to the base-case analysis (USD 3,464,658 at the national level and USD 0.0056 PMPM in the fifth year of change).

## 4 Discussion

This study demonstrated that changing the current reimbursement criteria for statin treatment among patients with T2DM by lowering the LDL-C threshold from 100 mg/dL to 70 mg/dL was associated with a reduction in CV events and related costs. The change in reimbursement criteria allowed an additional 1,011,770 patients with T2DM to be reimbursed for statin treatment, corresponding to 23% of all patients with T2DM and 2% of the entire population in South Korea. By using statins in these patients, we could expect 726 CV events to be prevented and USD 5.5 million to be saved at the national level in terms of annual disease-related costs.

A previous systematic review and meta-analysis showed that LDL-C reduction was associated with a decrease in the risk of major CV events by 19% per 1 mmol/L (Wang et al., 2020). The effect of LDL-C reduction was consistent across various baseline LDL-C levels, and patients with LDL-C <80 mg/dL showed a 17% risk reduction per 1 mmol/L. This finding was also consistent in both diabetes and non-diabetes patients (relative risk [RR] = 0.83, 95% confidence interval [CI] = 0.79–0.88 in diabetes patients; RR = 0.84, 95% CI = 0.78–0.90 in non-diabetes patients). Therefore, the study suggested a benefit for LDL-C management regardless of patient baseline LDL-C levels, even in patients with relatively low LDL-C levels who are generally not treated with lipid-lowering therapies.

In the Asian population, the EMPATHY study suggested the benefit of treating patients with intensive goals (LDL-C <70 mg/dL) compared to that in the standard goal (LDL-C between 100 and 120 mg/dL) among patients with diabetic retinopathy and hypercholesterolemia (Itoh et al., 2018). Although the primary outcome (i.e., CV events and CV-related deaths) did not show statistical significance (hazard ratio [HR] = 0.84, 95% CI = 0.67–1.07), cerebral events were significantly decreased in the intensive group (HR = 0.52, 95% CI = 0.31–0.88). In the sub-analysis of the EMPATHY study, in patients with blood pressure ≥130/80 mmHg, the intensive goal was associated with a significant decrease in the risk of the primary outcome compared to that in the standard goal (HR = 0.70, *p*-value = 0.015), whereas there was no significant association in patients with blood pressure <130/80 mmHg (Shinohara et al., 2021). These findings suggest that an intensive treatment goal is advantageous in Asian populations with T2DM, especially in patients with additional risk factors such as elevated blood pressure.

In most countries, statin treatment is recommended based on baseline risk assessment considering various patient risk factors. For example, the National Institute for Health and Care Excellence in the United Kingdom recommends patients with T2DM to use atorvastatin 20 mg if they have a ≥10% 10-years risk of CVD, as assessed by the QRISK2 risk calculator (National Institute for Health and Care Excellence, 2014). In contrast, the Korean guidelines for the management of dyslipidemia still recommend the use of LDL-C goals because of the lack of evidence regarding CV risk prediction and the impact of high- or moderate-intensity statin treatment in Asian patients (Kim et al., 2016). In a recent study exploring the association between LDL-C levels and the risk of CVD in Korean patients with T2DM who had no history of CVD and were treated with statins, the risk of myocardial infarction and stroke was significantly increased in those with an LDL-C level of 70–99 mg/dL compared to those with an LDL-C level <70 mg/dL (HR = 1.07, 95% CI = 1.03–1.12 for myocardial infarction; HR = 1.07, 95% CI = 1.03–1.11 for stroke) (Kim et al., 2019). This suggests the unmet needs for the prevention of CV events among patients with T2DM with LDL-C levels between 70 and 100 mg/dL. Further studies on CVD risk and statin effectiveness in the Korean population are warranted, and recent evidence from the Korean population should be considered in the lipid management of patients with T2DM.

Despite of the guidelines on CVD prevention developed by the European Society of Cardiology (ESC), two large-scale cross-sectional surveys in European countries revealed that a large proportion of patients with CHD received suboptimal management for CVD risk factors (Kotseva et al., 2009; Kotseva et al., 2016; Piepoli et al., 2016). Optimizing the management of CVD risk factors in these patients, such as using statins to achieve the LDL-C goals and smoking cessation, was found to be cost-effective (De Smedt et al., 2012; De Smedt et al., 2018). Our study also suggested that the CVD risk management at a national level can lead to decrease in the costs of CVD. To relieve the global burden of CVD, proactive strategies for the CV risk management need to be sought by considering the country-specific healthcare policies.

The findings of this study can be generalized to the Korean population by using Korea-specific data as extensively as possible. In particular, we utilized two types of real-world big data: the NHIS-HEALS and NHIS Customized databases. The NHIS-HEALS database is a representative sample of all patients who participated in health screening, thereby ensuring the representativeness of the clinical laboratory data. The NHIS Customized database contains patients of interest extracted from the entire Korean population. Therefore, the results obtained from the database can be generalized to the entire population. We also verified the appropriateness of the patient definitions and assumptions used in the model through expert elicitation.

However, the results should be interpreted with caution due to several limitations. First, we assumed that the proportion of atorvastatin prescriptions in patients with LDL-C between 70 and 100 mg/dL would be the same as that in patients with LDL-C ≥100 mg/dL because there was no evidence of the proportion of atorvastatin prescription in patients with T2DM with LDL-C between 70 and 100 mg/dL under the current reimbursement criteria. We also considered that the proportion of atorvastatin prescriptions in patients with LDL-C between 70 and 100 mg/dL would not exceed that in patients with LDL-C ≥100 mg/dL and therefore assumed a proportion between 0 and 11.6%. This assumption is partly supported by expert elicitation. Second, the efficacy of statin treatment was not specific to the Korean population owing to a lack of evidence. Third, although we matched patient groups by propensity scores when estimating baseline CV event rates, unmeasured confounders might exist. Lastly, the baseline LDL-C levels used in this study were derived from biennial health screening data, and therefore the levels might vary from the actual LDL-C levels at the initiation of statin treatment. However, we defined the baseline LDL-C level as the most recent value measured before the start of statin treatment. Fourth, the databases used in this study did not include the entire patients with diabetes in Korea, which might limit the generalizability of our findings. Fifth, the efficacy of statins in the primary prevention of CV events might vary depending on the baseline LDL-C level. However, in a previous meta-analysis conducted by the Cholesterol Treatment Trialists’ Collaborators, there was no difference in the effects of statin therapy among subgroups having different baseline LDL-C levels (Cholesterol Treatment Trialists' Collaborators et al., 2008).

## 5 Conclusion

In patients with T2DM without a history of CV events, easing the current reimbursement criteria for statin treatment by lowering the LDL-C threshold from 100 mg/dL to 70 mg/dL was associated with a reduction in CV events and related costs in South Korea. Changing the reimbursement criteria is likely to have an additional benefit in terms of the clinical and economic burden of CVD in patients with T2DM and therefore requires further consideration by policymakers.

## Data Availability

The datasets presented in this article are not readily available because this study data was extracted and analyzed from the National Health Insurance Service (NHIS) claims database, and additional data may be obtained from a third party (with appropriate authorization approval) but are not publicly available. Requests to access the datasets should be directed to National Health Insurance Sharing Service, https://nhiss.nhis.or.kr/
